# Recovery of Plastics from WEEE through Green Sink–Float Treatment

**DOI:** 10.3390/ma17123041

**Published:** 2024-06-20

**Authors:** Annarita Fiorente, Germano D’Agostino, Andrea Petrella, Francesco Todaro, Michele Notarnicola

**Affiliations:** Department of Civil, Environmental, Land, Building Engineering and Chemistry (DICATECh), Polytechnic University of Bari, Via E. Orabona n. 4, 70125 Bari, Italy; annarita.fiorente@poliba.it (A.F.);

**Keywords:** WEEE, sink–float, recycling, sustainability, plastics

## Abstract

Increasing demand for electrical and electronic equipment results in the generation of a rapidly growing waste stream, known by the acronym WEEE (waste electrical and electronic equipment). The purpose of this study was to evaluate the effectiveness of green sink–float treatment in sorting plastic polymers typically found in WEEE (PP, ABS, PA6, PS, and PVC). Molasses, a by-product of sugar bio-refining, was added in various concentrations to water to form solutions at different densities. The methodology was initially tested on virgin polymers; later, it was applied to plastics from a WEEE treatment plant. The polymers were characterised through near infrared spectroscopy (NIRS) and Fourier-transform infrared spectroscopy (FTIRS) analyses; the detection of any additives and flame retardants was conducted using the sliding spark technology (SSS2) and scanning electron microscope (SEM—EDX). The results showed that, for plastics from WEEE, the recovery efficiency was 55.85% for PP in a solution of tap water while the remaining part of PP (44.15%) was recovered in a solution of water to which 90% molasses was added. Furthermore, 100% recovery efficiency was obtained for PS and 93.73% for ABS in a solution of tap water with the addition of 10% *w*/*v* molasses. A recovery efficiency of 100% was obtained for PVC and 100% for PA6 in a solution consisting solely of molasses.

## 1. Introduction

Waste electrical and electronic equipment (WEEE) comprises a wide range of devices that use electricity, including all components and materials that are an integral part of them, which the owner intends to discard as faulty, obsolete, or unused [1].

Such equipment represents the fastest-growing waste stream in the world due to the advancement of technological innovation and the availability of new products on the market at affordable prices [2]. Approximately 62 billion kg of WEEE was generated globally in 2022 and this figure is expected to grow; in fact, it is estimated that in 2030, global generation will be 82 billion kg of e-waste [3].

WEEE consists of ferrous and non-ferrous metals (copper and aluminium), plastics, glass, precious materials, and rare earth that, if properly treated, can be recovered and reintroduced into the economic cycle [4]. However, hazardous substances (such as mercury, lead, ozone-reducing substances, brominated flame retardants, etc.) are present in some equipment, which, if not properly managed in the recycling process, can cause environmental impacts and harm to human health [5].

Directive 2012/19/EU sets high collection and recycling targets for Member States. As of 2019, the minimum collection rate required has been 65% of the average weight of EEE put on the market in the preceding 3 years or 85% of the weight of WEEE generated annually in each Member State [1]. By 2022, only 42.8 percent of the e-waste generated in Europe was collected and recycled properly [3]. Therefore, effective recycling strategies are sorely needed to promote the proper treatment of this waste and an environmentally sound recovery of secondary resources.

In Italy, WEEE from private households is classified into five groupings: R1—Cold and climate; R2—Large white goods; R3—Appliances with screens; R4—Consumer electronics; and R5—Light sources [6].

The R2 grouping, which includes large household appliances (i.e., dishwashers, washing machines, ovens, hobs, and electric stoves), is the category with the highest amount of waste generated and collected (about 35% by weight of total collection).

The average composition of WEEE belonging to waste group R2 is as follows: ferrous metals are the prevailing fraction with an average percentage value of about 50%; the share of plastics is around 13% while glass and non-ferrous metals are about 6% and 3%, respectively; a high fraction (around 30%) is represented by other materials (cement, rubber, wood, ceramics, etc.) [7].

The typical treatment cycle of WEEE in Group R2 includes as a first step the preliminary dismantling of hazardous and non-hazardous components, after which the carcasses are shredded and subjected to magnetic separation to remove ferrous metals. Next, the eddy current separator separates the non-ferrous metals from the plastics [8].

Although there are established approaches for the recovery of metal fractions, the recycling of the plastic component poses a significant challenge [9]. Indeed, this fraction is made up of numerous types of polymers that need to be appropriately sorted and separated in order to be reintroduced into the economic cycle. The main types of plastics that constitute WEEE in the R2 grouping are 56% PP (polypropylene); 7.8% PS (polystyrene); 3.3% ABS (acrylonitrile-butadiene-styrene); 4.7% PVC (polyvinylchloride); and 8% PA6 (polyamide 6) [10,11,12].

In addition, some plastics contain additives, such as flame retardants, including polybrominated biphenyls and diphenyl ethers (PBBs and PBDEs), which, in addition to being hazardous substances for the environment and human health, can alter the mechanical properties of the material (including density). This increases the complexity of the recovery of plastics from WEEE, favouring their disposal in landfills [13].

The Restriction of Hazardous Substances (RoHS) Directive (2011/65/EC) introduced threshold values for PBBs and PBDEs and, as a result, the presence of these hazardous substances in electrical and electronic equipment has been restricted. Maximum tolerated concentrations are set at 0.1% by weight in homogeneous materials, thus helping to promote the production of safer electronic devices and reduce the environmental impact associated with these substances [14]. However, household appliances, made of plastics in which brominated flame retardants are present, are still processed and it is, therefore, important to identify the best strategies to recover this product fraction [13].

The objective of this study is to analyse the effectiveness of the sink–float technique in sorting the different plastic polymers present in WEEE from the R2 grouping. The process exploits the difference in the density of materials, which, when placed in a solution, separate, as lighter ones tend to float (such as PP) while heavier (such as PVC) ones sink [15].

At the present time, several studies have been conducted involving the testing of the sink–float technique for the separation of different plastic polymers. In the study by Pongstabodee et al., 2007 [16], complete separation of HDPE from PP was achieved with 50% *v*/*v* ethanol(C_2_H_6_O) added in water. Separation of a PS/ABS mixture from a PET/PVC mixture was achieved using a 30% *w*/*v* solution of calcium chloride (CaCl_2_). Bill et al., 2022 [17] used solutions prepared with demineralised water and magnesium sulfate (MgSO_4_) at different concentrations for sorting different plastic polymers. In the study by Meneses Quelal et al. [18] the separation efficiency of plastics in aqueous solutions to which ethanol (C_2_H_6_O) and sodium chloride (NaCl) were added at different concentrations was evaluated, obtaining recovery rates ranging from 70% to 99.70% for virgin plastics.

In this study, with a view to sustainability, experiments were conducted using a solution of tap water (H_2_O) with the addition of sugar cane molasses at different concentrations. Molasses is a by-product obtained in the sugar production process that is in the form of a thick, brown-coloured liquid. The output product from a sugar bio-refining plant containing 46% sucrose, 28% water, about 19% nitrogenous substances, and about 7% mineral substances was used (values in line with data reported in the literature) [19]. Several studies used cane molasses for ethanolic fermentation because cane molasses can supply required nutrients for microorganism metabolism [20,21,22]. However, there is a need for additional technologies to recycle cane molasses in an environmentally friendly way [22,23].

The tests were conducted considering two types of mixtures: one consisting of virgin polymers typically found in WEEE in the R2 grouping (PP, ABS, PA6, PS, and PVC) and another consisting of polymers from a treatment plant. Both samples were subjected to detailed characterisation. NIRS and FTIRS were used to identify the polymers; sliding spark (SSS2) technology and scanning electron microscope (SEM) were used to detect the possible presence of brominated flame retardants and other additives in the plastics.

The purpose of this research is to find a suitable technology to recover plastics from WEEE through a sink–float treatment.

## 2. Materials and Methods

### 2.1. Characterisation of Plastic Polymers

The selected samples were characterised using the MiroSpark (IoSys, Ratingen, Germany), which was a combination of the NIRS and the SSS2. The NIRS was used to determine the composition and the type of polymers constituting the sample in percentages: the NIR gun was brought close to each non-black plastic particle and the result was displayed on the instrument screen. The SSS2, on the other hand, was used for the identification of black plastics.

In order to confirm the results obtained using the NIRS, analytical characterisation of the plastic polymers was performed using FTIRS. Specifically, the FTIRS Spectrum Two (PerkinElmer, Waltham, MA, USA) with an attenuated total reflection (ATR) diamond crystal accessory was used. Granules ranging in size from 2.5 to 5 mm were selected. Each particle was placed in contact with the diamond crystal accessory and the FTIR spectrum was acquired in transmittance with a wave number between 4000 and 400 cm^−1^. This evaluation was also performed on the virgin polymers previously considered in order to compare the absorption peaks of the infrared radiation of plastic granules from WEEE with virgin ones.

The identification of any additives in plastics was carried out using the MiroSpark sliding spark spectrometer (SSS2). The gun was moved closer to each sample and the results were displayed on the instrument’s display with a measurement time of 1 s. The measurement was repeated three times for each sample, and the plastics that showed the presence of additives and flame retardants were selected. In order to perform a more in-depth analysis, SEM coupled with energy-dispersive X-ray microanalysis (EDX) was used.

Three samples of ABS and three of PP, measuring 1.5 cm × 1.5 cm × 0.5 cm, were studied using an electron microscope FESEM-EDX 300 VP (Carl Zeiss Microscopy GmbH, München, Germany). All samples were metallised with gold for electrical conduction.

### 2.2. Sink–Float Technique for Virgin Plastic Polymers

The sink–float methodology was tested for the separation of virgin plastic polymers to the calibration of a recovery procedure to be applied to plastics from a WEEE treatment plant.

The sample consisted of six different types of virgin plastic polymers (typically found in R2 WEEE) in granular form: PP, ABS, PA6, PS, and PVC. Table 1 shows the densities of the materials considered [24].

Granular samples of 5 g of each virgin polymer (with particle sizes between 2.5 mm and 5 mm) were placed in a 1 L capacity beaker filled with 0.5 L tap water. Mechanical agitation was performed at a speed of 300 rpm using a flocculator equipped with adjustable rods and considering a mixing time (tm) of 5 min and a settling time (ts) of 10 min. Polymers with a density lower than tap water (PP and a very low amount of ABS) floated to the surface while particles with a higher density (PVC, PA6, ABS, PS) sank completely (Stage 1). The floated and precipitated samples were washed and dried at room temperature. Next, they were weighed and sorted manually to evaluate their composition.

**Figure 1:**
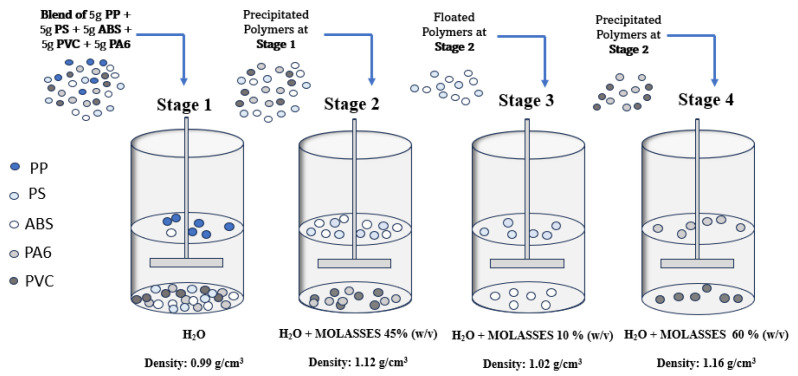
Sink–float methodology applied to virgin polymers.

Polymers with higher density than tap water were placed in a beaker filled with a solution of water and molasses (45% *w*/*v*) and the complete separation of PS and ABS from PVC and PA6 samples was verified (Stage 2). The former were sorted using a solution of water and molasses (10% *w*/*v*) (Stage 3). The latter were separated using a solution of water and molasses (60% *w*/*v*) (Stage 4) (Figure 1).

Molasses percentages were obtained through experiments aimed at optimising separation processes. The density of the solution was increased in a controlled manner by iteratively adding 5% *w*/*v* molasses to the 0.5 L aqueous solution. After each step, the solution was mechanically agitated using the test jar by a rotating propeller at 300 rpm for a time of 5 min. The objective of agitation is to ensure the homogeneity of the solution. After this, the density of the solution was assessed using a graduated cylinder in order to calculate the volume and mass of the solution. The polymer separation process was evaluated by iteratively adding 5% *w*/*v* molasses to the solution in order to determine the optimal concentration that guaranteed the highest recovery rate for each polymer. All tests were conducted in triplicate.

Results obtained from the application of the sink–float technique to polymer separation are expressed in terms of the recovery efficiency (RE) for each polymer. This parameter represents the ratio of the mass of the recovered polymer (*mr*) to its mass in the initial feed stream (*mf*), providing a quantitative indicator of the effectiveness of the separation procedure. When the mixture consisted of several polymers, the term selection coefficient (SC) was used:

(1) $$\mathrm{RE}=\frac{mr}{mf}\;✕\;100$$

### 2.3. Sink–Float Technique for Waste Plastic Polymers

A sample consisting of the plastic polymers from a WEEE R2 treatment plant in the percentages identified during characterisation was considered. Specifically, the predominant fraction was PP (12.3 g), followed by ABS (3.87 g), PS (2 g), PVC (2.14 g), and PA6 (2.08 g).

The test was carried out using the same process conditions as the experiment performed with virgin polymers (plastic particle size: 2.5–5 mm; flocculation speed: 300 rpm; a tm: 5 min; and a ts: 10 min).

The evaluation of the molasses percentages to be added to the solution was also carried out for plastics from WEEE in order to optimise their separation.

**Figure 2:**
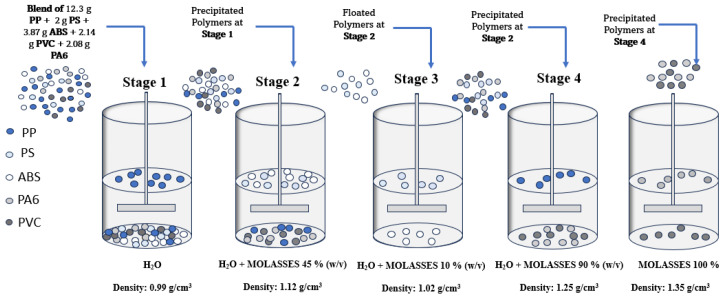
Sink–float methodology applied to polymers from WEEE treatment plant.

The plastics mixture was placed in a beaker containing only tap water (H_2_O). At this stage, the light fraction, mainly composed of polypropylene (PP) floated while the remaining part precipitated (Stage 1). The latter was placed in a solution of water and molasses (45% *w*/*v*). The PS and ABS polymers floated while PVC, PA6, and part of PP sank (Stage 2). Complete separation of PS from ABS was achieved in a solution of water and molasses (10% *w*/*v*) (Stage 3). By increasing the molasses concentration (90% *w*/*v*), the separation of polypropylene (PP) from the other polymers in the mixture (PA6 and PVC) was achieved (Stage 4). Separation of PA6 and PVC was achieved in a solution consisting of molasses (100%) (Stage 5). The process is shown in Figure 2.

## 3. Results

### 3.1. Characterisation of Plastic Polymers

**Figure 3:**
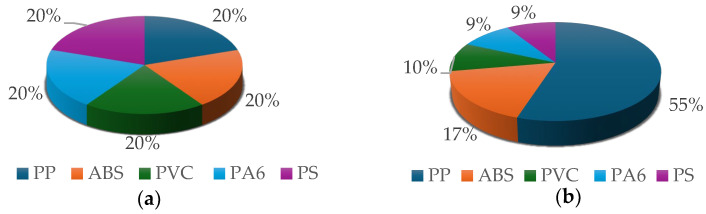
(**a**) Composition of the sample of virgin plastics; (**b**) Composition of plastics from WEEE.

Figure 3a shows the composition of the sample of virgin plastics. Analysis of the commodity composition of the sample of plastics leaving a WEEE R2 treatment plant, performed with the MiroSpark laboratory instrument, showed that the predominant plastic polymer is polypropylene (PP), which accounts for about 55% of the total fraction. ABS accounted for 17.28%, followed by PVC accounting for about 10% and PA6 and PS accounting for about 9%—Figure 3b.

**Figure 4:**
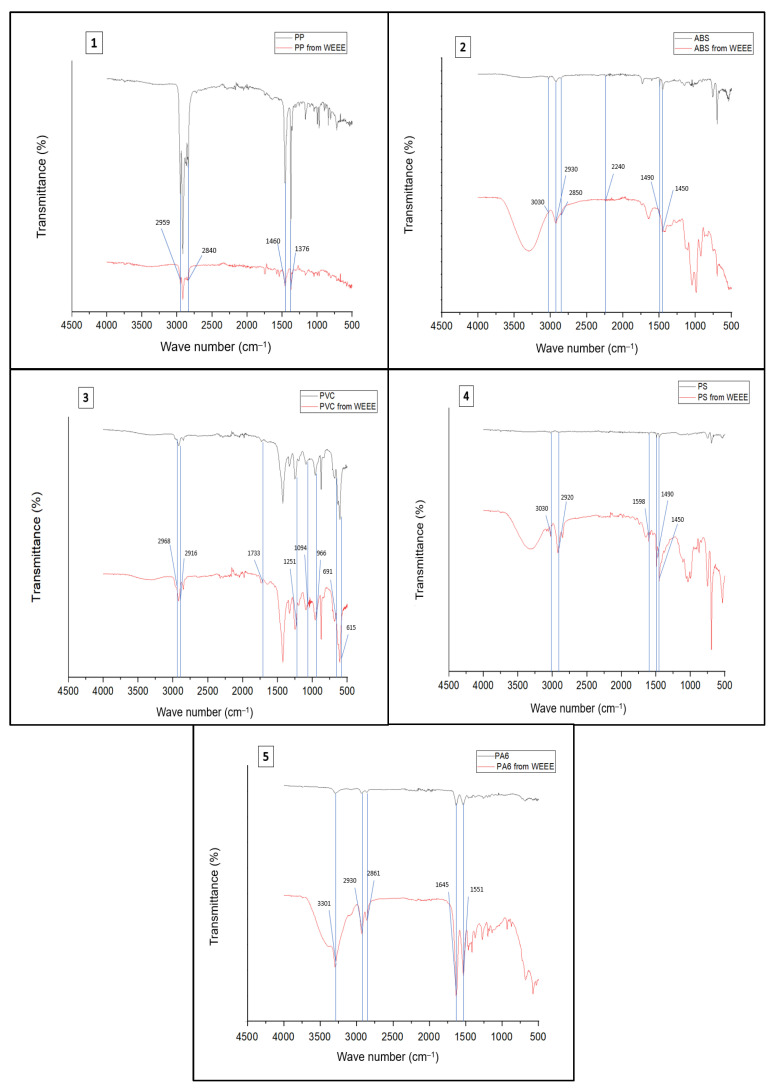
FTIR spectra of representative samples of plastic polymers in R2 WEEE: (1) PP, (2) ABS, (3) PVC, (4) PS e, (5) PA6. The black line refers to virgin polymers and the red line to polymers from WEEE.

The FTIR-ATR analysis allowed a more detailed identification of the polymer composition of the plastic fraction. Figure 4 compares the spectra of the analysed plastics from WEEE (red line) with those of the corresponding virgin polymers (black line).

The IR spectrum was analysed considering the absorption peaks related to the functional groups of the different materials analysed.

The spectrum of PP is represented by the absorption bands at 2959 and 2840 cm^−1^, due to the asymmetric stretching of the CH_3_ group and the symmetric stretching of the CH_2_ group, respectively. The absorption peaks at wave numbers of 1460–1376 cm^−1^ are related to the asymmetric stretching of the CH_3_ group and the symmetric stretching of the CH_2_ group. The corresponding functional group is the aliphatic CH [9].

The FTIR spectrum of ABS shows a characteristic absorption peak at 2240 cm^−1^, which is attributed to the nitrile group C≡N. The absorptions at 1450 and 1490 cm^−1^ are related to the CH_3_ group deformation. The absorption bands at 2850 cm^−1^ and 2930 cm^−1^ are attributed to the vibration of the aliphatic C-H bond while the peak at 3022 cm^−1^ is related to the aromatic C-H vibration [25].

The FTIR spectrum of PVC is characterised by absorption bands at 2968 and 2916 cm^−1^ related to C-H stretches, 1733 cm^−1^ attributed to C=O, 1251 cm^−1^, 1094 cm^−1^ related to the C-O extension coupled with the C-C vibration, 966 cm^−1^ attributed to skeletal vibrations, and 691 and 615 cm^−1^ attributed to C-Cl stretches and skeletal vibrations [25].

PS is characterised by absorption bands at 3030 and 2920 cm^−1^ for aromatic and aliphatic C-H group vibrations, respectively. The peak at 1598 cm^−1^ is representative of the vibration of the aromatic C=C group while the absorptions at 1490 and 1450 cm^−1^ represent the deformation of the aliphatic C-H group [9].

IR measurement results for (PA6) show an absorption band at 3301 cm^−1^, usually attributed to the N-H folding vibration in primary amines. The two bands at 2930 and 2861 cm^−1^ are attributed to the CH elongation of the ethylene sequence. In addition, the absorbance at 1645 cm^−1^ related to the C=O amide I extension and the combined absorbance of the N-H and C-N amide II vibration at 1551 cm^−1^ are clearly observed [26].

Figure 4 indicates the differences between the spectra of virgin polymers and the spectra of polymers from WEEE. Generally, the oxidation of PP is measured by quantifying the carbonyl region in the range of 1650–1850 cm^−1^ after placing the material in a high-temperature oven. The formation of asymmetric elongation C=O at 1765 cm^−1^ indicates the oxidation of PP from WEEE and, thus, degradation during the useful life of the polymer [27]. In the spectrum related to ABS from WEEE, the increased absorptions in the hydroxyl (3650–3200 cm^−1^) and carbonyl (1850–1650 cm^−1^) are clearly visible, indicating the degradation of the material. In the absorption region (1400–1000 cm^−1^) of C-O stretching vibrations, an important increase in absorbance is observed, although the positions of the peaks are not clearly visible [28]. In the PVC from the WEEE spectrum, an increase in absorbance can be seen in the spectral region from 1900 to 1500 cm^−1^, which is an indication of carbonyl formation and conjugation [29]. The spectrum of PS from WEEE shows greater absorption in the 2800–3000 cm^−1^ region, which is characteristic of C-H elongation in methyl or methylene groups [30]. The oxidative degradation of PA6 from WEEE can be seen in the graph showing the increase in total carbonyls in the range 1695–1800 cm^−1^ [31].

**Figure 5:**
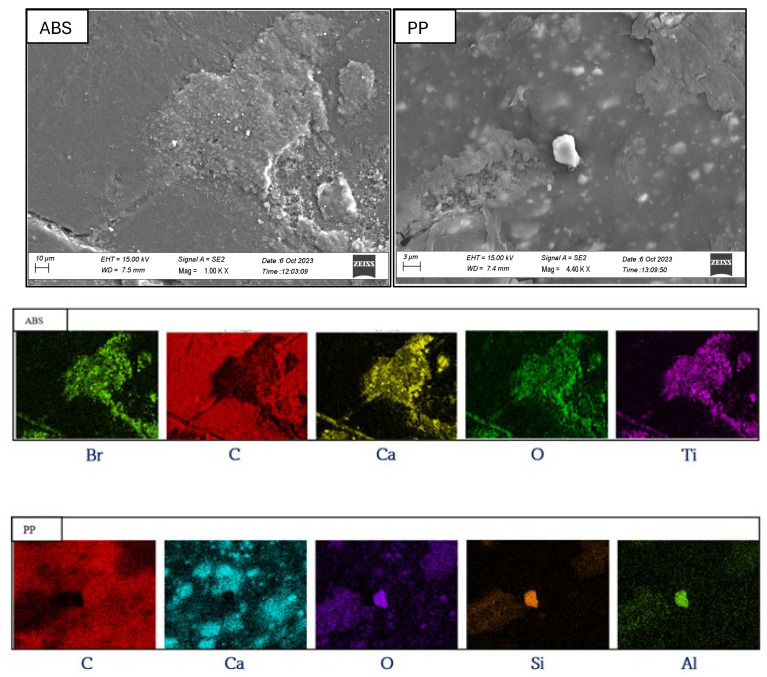
SEM images of ABS and PP plastic samples from WEEE with distribution maps.

The results of the analysis using SSS2 showed that virgin plastics had no significant amounts of additives or flame retardants. Regarding plastics from WEEE, PP-containing additives accounted for 18% of the total PP while ABS in which FR or additives were present constituted 26% of the total. No other elements were found in the other PS, PA6, and PVC plastics. The ABS and PP samples were subjected to scanning electron microscope analysis. Figure 5 shows SEM images at magnifications of 1000× and 4400× representative of the analysed samples.

Associated with these images are maps showing the distribution and accumulation areas of some of the chemical elements found in the most significant quantities by EDX microanalysis.

The results of the EDX microanalysis shown in Table 2 showed that the ABS plastic samples contained a bromine (Br) concentration of 1.08 (wt%), in contrast to the PP plastic samples where no significant amount of Br (%) was present.

These data reveal the possible presence of brominated flame retardants (BFRs) in the ABS; in the PP plastic samples, the 1.78 (wt%) of aluminium (Al) could indicate the presence of non-halogenated flame retardants and smoke suppressants, such as Al (OH)_2_ × 3H_2_O)/AlOOH × H_2_O. The results for Ti (%) showed that the ABS plastic samples likely contained the pigment TiO_2_, which was used as a dye and UV stabiliser [32], while it is admissible that Zn was used in the PP plastic samples as a stabiliser. The presence of magnesium (Mg) of 2.69 (wt%) and silicon (Si) of 7.17(wt%) was identified in the PP samples, which can be attributed to talc (H_2_Mg_3_(SiO_3_)_4_), a filler typically used in this polymer [32]. Ca (%) is present in both polymers with a good probability attributable to CaO, often used as an opacifying agent in plastics [24].

### 3.2. Sink–Float Technique for Virgin Plastic Polymers

**Figure 6:**
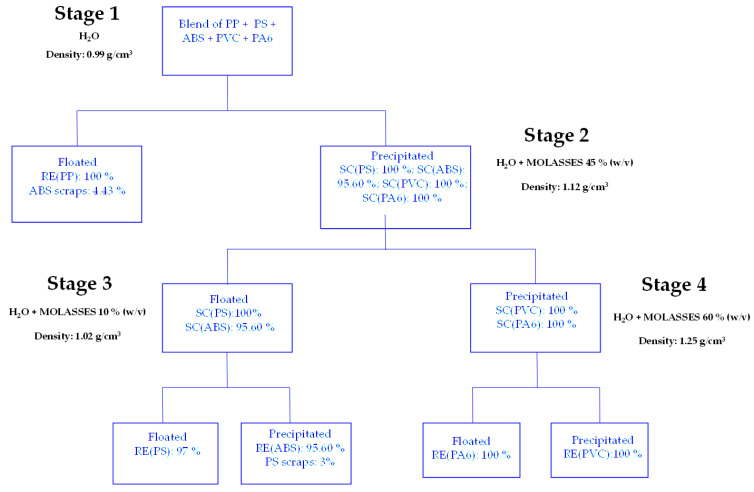
Flow chart of virgin polymers recovery by the sink–float method.

Using only tap water (H_2_O), it is possible to achieve 100% recovery efficiency of PP (Stage 1). By using a solution composed of water and molasses (45% *w*/*v*) (Stage 2), it is possible to achieve the separation of the mixture precipitated in the first stage. Specifically, PS and ABS floated and PVC and PA6 precipitated. In order to further separate PS from ABS, a solution consisting of tap water and molasses (10% *w*/*v*) is considered (Step 3). This results in the recovery of 97% of PS that floats and 95.60% of ABS that precipitates. By placing the mixture of PVC and PA6 in a solution of tap water and molasses (60%) (Step 4), the recovery of 100% of PA6 that floats and 100% of PVC that precipitates is obtained (Figure 6).

### 3.3. Sink–Float Technique for Waste Plastic Polymers

**Figure 7:**
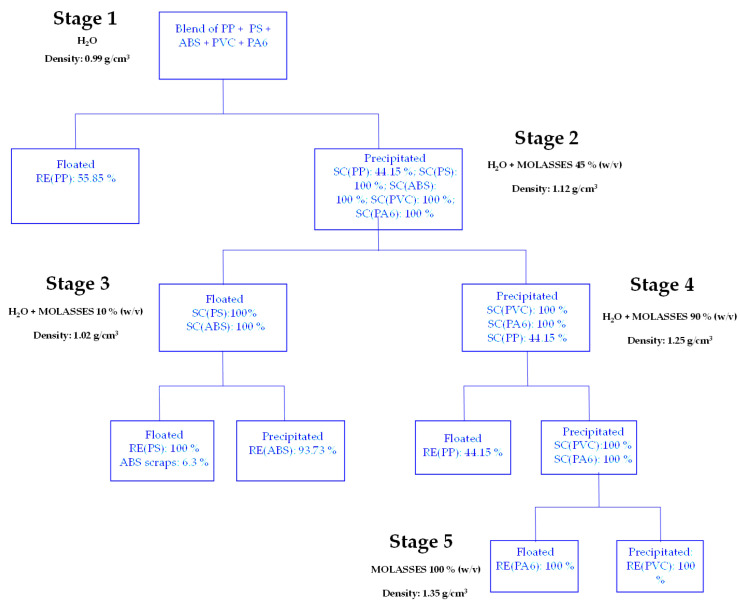
Flow chart of the plastics from WEEE recovery by the sink–float method.

In order to recover the plastic polymers PP, PS, ABS and PVC, and PA6 constituting the output plastic fraction from a WEEE R2 treatment plant, an aqueous solution is used, to which molasses is added in various concentrations (Figure 7).

In Stage 1, through the exclusive use of tap water (H_2_O), a recovery of 55.85% is achieved for polypropylene (PP). Considering a solution consisting of water and a molasses concentration of 45% *w*/*v* (Phase 2), the separation of the polymers that precipitate in Stage 1 is achieved: the total amounts of PS and ABS float on the surface while 44.15% PP, 100% PA6, and 100% PVC sink to the bottom. The mixture floated in Stage 2 (ABS + PS) is placed in a tap water solution to which 10% *w*/*v* molasses is added: 100% recovery of PS and 93.73% of ABS is thus obtained. The sample precipitated in Phase 2 (PVC, PA6, PP) is placed in a tap water solution to which 90% *w*/*v* molasses is added: thus, complete separation of the PP that floats from the mixture of PVC and PA6 that precipitates is obtained. By using a molasses solution (100% *w*/*v*), complete separation of PA6 from PVC is achieved.

## 4. Discussion

The results show that solution density is an important parameter in the sink–float technique as it determines the effectiveness of the methodology in recovering plastic polymers in R2 WEEE.

The decision to use solutions of water and molasses in various concentrations is related to the fact that this is a natural product obtained from the bio-refining of sugar, which, in addition to having no impact on the environment, is also very effective in separating the different polymers. In fact, this is characterised by a high density (1.35 g/cm^3^), which allows the preparation of solutions capable of selecting even the heaviest polymers: using a solution consisting exclusively of molasses (100%), it is possible to achieve a recovery efficiency of 100% of PA6 and PVC from WEEE. The other solutes typically used in the sink–float process (NaCl, MgSO_4_) do not have a high solubility in water and do not allow solutions to be obtained that would allow the selection of heavier polymers. For example, the maximum amount of sodium chloride (NaCl) that can be added to water before saturation is 180 g (36% *w*/*v*) [33], allowing for a solution having a density of 1.16 g/cm^3^.

**Figure 8:**
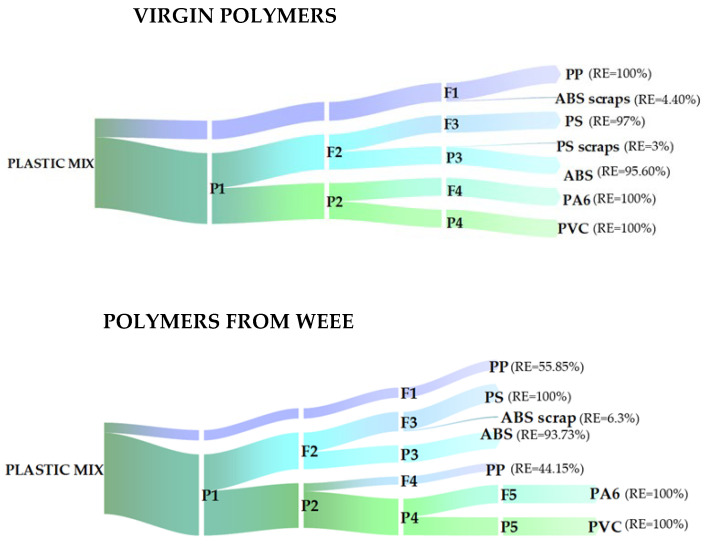
Results obtained from the sink–float process applied to virgin polymers and polymers from WEEE in terms of recovery efficiency.

Figure 8 shows the results obtained by applying the sink–float methodology to virgin plastic polymers and polymers from a WEEE R2 treatment plant and the recovery efficiencies are highlighted. Differences in the behaviour of the plastics emerge, which can be attributed to the fact that those from the WEEE R2 treatment plant may contain additives and flame retardants that influence their density [34].

Virgin polypropylene (PP) is a polymer with a density in the range of 0.87–0.92 g/cm^3^; in fact, it floats in a solution of tap water only (density: 0.99 g/cm^3^), resulting in 100% recovery. The same polymer from the WEEE R2 treatment plant shows a different behaviour: only 55.85% floats in tap water and, therefore, has a lower density than that of the solution; the remaining part (44.15%) precipitates in a 45% (*w*/*v*) molasses solution with a density of 1.12 g/cm^3^ but floats in a 90% (*w*/*v*) molasses solution with a density of 1.25 g/cm^3^. It can therefore be deduced that the polymer has a specific gravity between the two values.

This result can be interpreted with reference to the analyses conducted during characterisation. In fact, identification with the SSS2 shows that about 18% of PP has additives. Furthermore, characterisation with the SEM shows the presence of 2.6 (wt%) magnesium and 7.17 (wt%) silicon in the samples of this polymer, which can be attributed to the presence of talc (H_2_Mg_3_(SiO_3_)_4_). Typically, this additive in the PP matrix is present in the range of 10 to 40% (wt%), providing the plastic with higher stiffness, better surface aesthetics, a lower coefficient of thermal expansion, and greater resistance to scratching and thermal deformation [24].

However, the addition of this filler results in an increase in polymer density. Referring to that of virgin PP and its behaviour in the sink–float application, it is possible to estimate that 44.15% of PP from WEEE, with a density between 1.12 g/cm^3^ and 1.25 g/cm^3^, is characterised by the addition of about 40% talc by weight. The increase in specific weight could also be caused by other additives, such as the non-halogenated flame retardants Al (OH)_2_ × 3H_2_O)/AlOOH × H_2_O; in fact, 1.78 (wt%) aluminium is found in the polypropylene samples [32].

Acrylonitrile butadiene styrene (ABS) from WEEE exhibits similar behaviour to virgin polymers, although the presence of possible additives and flame retardants is found in 26% of the total polymer characterised. In scanning electron microscope (SEM) analysis, it is observed that bromine (Br) constitutes 1.08 (wt%) of the material, suggesting the possible presence of brominated flame retardants (BRF) in this plastic. Furthermore, titanium (Ti) accounts for 8.57% (wt%) and could indicate the presence of TiO_2_, used as a dye and UV stabiliser [32]. Given the titanium content (incorporated into the polymer matrix of plastics to improve their functionality), the separation of titanium dioxide from ABS through chemical recycling techniques is desirable [35].

The complete separation of ABS from polystyrene (PS) is achieved in a 10% (*w*/*v*) molasses solution, resulting in a recovery of approximately 95% of the polymer.

Virgin PA6 floats in a 60% (*w*/*v*) molasses solution, allowing 100% recovery of the polymer. In contrast, PA6 from WEEE precipitates in a 90% (*w*/*v*) molasses solution having a density of 1.25 g/cm^3^. This result could be associated with the presence of glass fibres (30%) used to give the plastic good mechanical properties and high strength [36]. By using a molasses solution (100% *w*/*v*), complete separation of PA6 from PVC is achieved.

Polystyrene (PS) and PVC from an R2 waste electrical and electronic equipment (WEEE) treatment plant exhibit similar behaviour to the same virgin polymers when applying the sink–float technique.

Molasses, other than being a natural and green reagent, has proven to be effective in separating different polymers. It originates from the sugar refining process so it is not considered waste but a by-product, as it can be used directly without further treatment in subsequent production processes. Thanks to its high concentration of sugars, such as glucose, sucrose, and fructose, as well as nutritive minerals, it is widely used for fermentative ethanol production [20]. Many studies have also demonstrated the effectiveness of molasses as the sole carbon source for anaerobic digestion processes aimed at producing hydrogen and methane [37].

Compared to other technologies currently used in plastics recovery plants, such as NIR optical sorting and triboelectrostatic separation, sink–float technology has some distinctive advantages. Firstly, unlike NIR optical sorting, which is ineffective in identifying dark-coloured plastics due to the limitations of near-infrared spectral analysis, sink–float separation is unaffected by the colour properties of the materials. Compared to triboelectrostatic separation, sink–float technology could offer further advantages in separating plastics without additives and plastics with additives, which often have different densities [24]. In addition, the use of green reagents, such as molasses, would provide further benefits as the solution used for separation could be used as biomass for the sustainable production of energy carriers, such as biogas and hydrogen [37].

Preliminary analysis demonstrates the economic feasibility of the sink–float technique. This process makes it possible to obtain almost completely pure polymers, which therefore have a high market value.

The sink–float process on an industrial scale entails a significant cost of approximately EUR 400 per ton of processed material. This cost includes several essential components, such as the sink–float separator, the mechanical mixing system, a plastic washing and drying system, and a solution filtering process. In addition to the direct process costs, the operating costs include the electricity required for the mechanical agitation of the solution. One possible strategy to reduce these costs is the use of molasses as a reagent for the solution. [38]. Molasses could not only replace other more expensive reactive agents but could also be used for energy production. This approach could lead to an overall cost reduction of 20%, thus improving the economic viability of the sink–float process on an industrial scale.

From an environmental point of view, the main benefit of separating plastic polymers lies in the fact that it avoids the energy consumption associated with the production of virgin polymers. In fact, the energy consumption of the sink–float process is negligible, making this technique a sustainable and environmentally friendly solution for recycling plastics [39].

## 5. Conclusions

In this study, the sink–float technique was used to separate virgin plastic polymers and those found in WEEE category R2 and the results were compared.

Characterisation of the plastics identified additives and flame retardants present in the polymers. The presence of significant amounts of additives in virgin plastics was not identified. Regarding the polymers from WEEE, talc was added to PP (about 40%), the ABS analysed contained small amounts of bromine (Br) and probably consisted of TiO_2_ used as a dye and UV stabiliser, and PA6 was reinforced with glass fibres.

The experimentation of the sink–float methodology was conducted using molasses, a by-product of the sugar production process, by which it was possible to achieve a solution with a high density, allowing the selection of heavier plastic polymers.

The presence of additives in polymers from WEEE has resulted in changes in their density and different behaviour in the sink–float technique compared to virgin polymers. By applying the sink–float technique first to virgin plastics and then to plastics from WEEE, it was possible to make a comparison and identify different behaviours of polymers containing additives.

However, when applied to plastics from WEEE, this methodology recovers 55.85% PP in a tap water solution, 100% PS, 93.73% ABS, and the remaining 44.15% PP in a molasses and water solution. By using a molasses solution (100% *w*/*v*), complete separation of PA6 from PVC is achieved.

The sink–float separation technique with molasses is an environmentally friendly, economical, and potentially effective method for separating and sorting plastic polymers from WEEE.

## Figures and Tables

**Table 1:** Density of virgin plastic polymers typically present in WEEE R2 [24].

Polymer	Units	Value
PP	g/cm^3^	0.87–0.92
PS	g/cm^3^	1.016–1.06
ABS	g/cm^3^	1.04–1.07
PA6	g/cm^3^	1.12–1.14
PVC	g/cm^3^	1.40–1.42

**Table 2:** Results of EDX microanalysis of WEEE plastic samples.

	C	O	Ti	Ca	Br	Fe	Al	Si	Others
	%	%	%	%	%	%	%	%	%
ABS	68.0 ± 5.4	17.6 ± 1.5	8.6 ± 0.8	2.9 ± 0.5	1.1 ± 0.3	0.6 ± 0.1	0.2 ± 0.1	0.2 ± 0.1	0.8 ± 0.1
PP	42.9 ± 2.5	29.3 ± 1.9	-	11.4 ± 0.5	-	1.1 ± 0.1	1.8 ± 0.1	7.2 ± 0.6	6.3 ± 0.5

## Data Availability

The original contributions presented in the study are included in the article, further inquiries can be directed to the corresponding author.
